# Polysaccharides from Chinese Herbal *Lycium barbarum* Induced Systemic and Local Immune Responses in H22 Tumor-Bearing Mice

**DOI:** 10.1155/2018/3431782

**Published:** 2018-06-03

**Authors:** Xiangliang Deng, Shuang Luo, Xia Luo, Minghua Hu, Fangli Ma, Yuanyuan Wang, Xiaoping Lai, Lian Zhou

**Affiliations:** ^1^School of Pharmaceutical Sciences, Guangzhou University of Chinese Medicine, Guangzhou 510006, China; ^2^Infinitus Chinese Herbal Immunity Research Centre, Guangzhou 510600, China; ^3^Dongguan Mathematical Engineering Academy of Chinese Medicine, Guangzhou University of Chinese Medicine, Dongguan 523000, China

## Abstract

*Lycium barbarum* polysaccharide (LBP) is isolated from the fruit of Chinese herbal *Lycium barbarum*. Previous studies had demonstrated that LBP could inhibit tumor growth and enhance the immunity in mice. However, the effect of LBP on systemic and local immune responses *in vivo*, especially on phenotypic and functional changes of T cells, is still largely unknown. In the present study, we investigated the effects of LBP on systemic and local T cell-dependent antitumor immune responses in H22 tumor-bearing mice. The results showed that LBP could inhibit the solid tumor growth in mice, but showed little effect on the body weight or spleen index. Furthermore, LBP could maintain high levels of T cells in peripheral blood (PB), tumor draining lymph node (TDLN), and tumor tissue, prevent the increase of Tregs while promote infiltration of CD8^+^ T cells in tumor tissue, inhibit the production of TGF-*β*1 and IL-10 in serum, decrease the exhaustion phenotype of T cells, and maintain cytotoxicity of lymphocytes. Taken together, our results demonstrated that LBP simultaneously induced systemic and local immune responses in H22 tumor-bearing mice by alleviating immunosuppression and maintaining antitumor immune responses in mice.

## 1. Introduction

*Lycium barbarum* polysaccharide (LBP) is isolated from the fruit of edible Chinese herbal *Lycium barbarum.* LBP has multiple biological activities and function, such as antitumor activity [1–3], immunoregulation [4–6], neuroprotective effect [7], and cardioprotective activity [8]. The antitumor activity of LBP had been demonstrated in the tumor-bearing mice that it could inhibit transplantable sarcoma S180 [2] and hepatoma H22 tumor growth in mice [1]. Furthermore, LBP could enhance the immunity of the tumor-bearing mice by improving lymphocyte proliferation and increasing macrophage phagocytosis and cytotoxic T lymphocyte (CTL) activity [1, 2]. However, the effects of LBP on systemic and local tumor immune responses are still largely unknown.

Cancer is a complex collection of distinct genetic diseases that it causes millions of deaths each year around the world [9, 10]. Over the past decades, with the development of understanding of the cellular and molecular mechanisms of immune system, the important roles of immune cells and molecules in cancer development and prevention have been identified and demonstrated. It is now clear that T cells as one of the major forces of adaptive immunity play a duplicitous role in cancer development—either pro- or antitumor growth due to different cell subsets [11, 12]. Evidence had accumulated that the presence of high levels of T cells, including CD8^+^ CTL and CD4^+^ helper T cell (Th cell), was a favourable prognostic factor in human tumors [13–15]. However, evidence showed that the increase of CD4^+^ Tregs indicated poor prognosis in tumor-bearing individuals [16–18]. Tregs can suppress antitumor responses of CD8^+^ CTL and CD4^+^ Th. It had been demonstrated in tumor-bearing mice that the depletion of Tregs could enhance antitumor immunity and inhibit tumor growth [19]. It is one of the most promising methods for cancer therapy to maintain an effective antitumor T-cell response in cancer patients. In this case, immunotherapy which activates the immune system to fight against cancer cells has become an effective approach in some cancer treatments.

Previous studies had reported that LBP could activate T cells [5, 20] and regulate the phenotypic and functional maturation of murine bone marrow-derived dendritic cells (DC) [6]. LBP-treated DC could improve Th1 and Th2 responses both *in vitro* and in *vivo* [21]. Another study led by Bo et al. showed that simple nanoliposomes encapsulating *Lycium barbarum* polysaccharides efficiently stimulated CD4^+^ and CD8^+^ T cell proliferation *in vivo* [4]. Furthermore, LBP showed synergistic immunotherapeutic effects when combined with interferon-*α*2b in murine rencarenal carcinoma treatment [22]. However, the effect of LBP on systemic and local immune responses *in vivo*, especially on phenotypic and functional changes of T cells, is still largely unknown. In our previous study, we found that a fraction from LBP had the highest antitumor activity in H22 tumor-bearing mice [23]. In the present study, we further investigated the effect of LBP on immune responses both in system and tumor tissue.

## 2. Materials and Methods

### 2.1. Reagents

LBP was prepared from *Lycium barbarum* by our laboratory as described previously [23]. The total sugar and protein content was 70.13% and 19.30%, respectively. The fractions with molecular weight range from 40 kDa to 350 kDa were prepared and used in this study. Mouse 1x lymphocyte separation medium was purchased from Dakewe Biotech Co. Ltd. (Shenzhen, China). PE/CY7-anti-mouse CD3, FITC-anti-mouse CD4, PE-anti-mouse PD-1, PE/CY5-anti-mouse CD25, and purified CD8 antibody were purchased from BioLegend. Purified CD3 antibody was purchased from Affinity Bioscience. Propidium iodide (PI), collagenase type IV, and DNase I were purchased from Sigma. Mouse IL-10 and mouse TGF-*β*1 ELISA kit were purchased from MULTI SCIENCE (Hangzhou, China).

### 2.2. Animals

Specific pathogen-free six-to-eight-week-old male BALB/c mice, weighed 20 ± 2 g, were purchased from the Guangdong Medical Laboratory Animal Center (Foshan, China). Animals were fed on standard laboratory diet and water, and all experimental procedures were approved by the Animal Care and Use Committee of Guangzhou University of Chinese Medicine, Guangzhou, China.

### 2.3. Preparation of Tumor-Bearing Mice and Treatment Protocol

H22 tumor-bearing mice were prepared as described previously [23]. Briefly, the tumor-bearing mice were prepared by being injected subcutaneously with 2 × 10^6^ H22 cells into the right armpit. The tumor-bearing mice were divided into model group and LBP treatment group, while tumor-free mice were used as control. The mice in LBP treatment group were treated intragastrically with 250 mg/kg LBP (dissolved in saline solution) for 10 days once daily since day 1 after tumor challenge. The control and model mice were given the same volume of saline solution intragastrically.

### 2.4. Analysis of Tumor Weight, Body Weight, and Spleen Index

The body weight of the mice was recorded every day. On day 11, mice were sacrificed by cervical dislocation after anaesthesia with chloral hydrate. The tumors and spleens were excised, photographed, and weighed. The spleen index was calculated using the formula: spleen index (mg/g) = spleen weight (mg)/body weight (g).

### 2.5. Preparation of Lymphocytes

Single cell suspensions of lymphocytes from PB, TDLN, spleen, and tumor tissue were prepared as described below. Lymphocytes from freshly heparinized PB were prepared with a mouse 1x lymphocyte separation medium (Dakewe Biotech Co. Ltd., China) according to the manufacturer's instructions. Lymphocytes from the spleens and TDLNs were prepared as described previously [24]. Briefly, the spleens and TDLNs were aseptically removed and ground gently by passing a sterile 200-gauge steel mesh. The cells were collected by centrifugation at 300 ×g for 5 min at 4°C. The lymphocytes were prepared with the lymphocyte separation medium. The cells were washed twice with precold phosphate buffer (PBS) and resuspended in PBS for flow cytometry analysis or in RPMI1640 medium (containing 10% FBS, 100 U/mL penicillin, and 100 *μ*g/mL streptomycin) for cytotoxicity analysis. To prepare lymphocytes from tumor tissues, tumors were cut into small pieces and digested with 25 *μ*g/mL collagenase type IV and 150 U/mL DNase I in RPMI1640 for 24 h at 4°C. Then, the tumor tissues were gently pressed through a sterile 200-gauge steel mesh with a plunger. Cells were collected by centrifugation at 300 ×g for 5 min at 4°C and washed twice with PBS. The cells were resuspended and taken to prepare lymphocytes with the mouse 1x lymphocyte separation medium as described above.

### 2.6. Flow Cytometry Analysis

Phenotypic analysis was performed by flow cytometry (FCM). The lymphocytes were stained with fluorescence-conjugated monoclonal antibodies as follows: PE/CY7-antimouse CD3, FITC-anti-mouse CD4, PE-anti-mouse PD-1, PE/CY5-anti-mouse CD25, or PE/CY5-anti-mouse CD8 according to the manufacturer's instructions. And then, the cells were detected at medium speed using a FACS Canto™ II flow cytometer (BD Biosciences).

### 2.7. ELISA Assay

Freshly heparinized blood was prepared from orbital venous plexus of the mice. After being centrifuged at 800 ×g for 20 min at 4°C, sera were collected. The levels of TGF-*β* and IL-10 in sera were assayed by enzyme-linked immunoabsorbent assay (ELISA) kits according to the manufacturer's instructions.

### 2.8. Cytotoxicity Analysis

The cytotoxicity of lymphocytes was detected as described previously with minor modification [2]. Briefly, H22 cells as target cells were labeled with 5 *μ*mol/L CFSE for 10 min at room temperature in the dark and washed thrice with RPMI1640 medium containing 5% FBS. The effector cells (lymphocytes) and target cells (H22 cells) were incubated in 96-well U-bottom plate for 24 h at a ratio of 50 : 1. The cells were collected and washed twice with PBS. After being stained of PI, cells were detected by FCM and the percentage of CFSE^+^ PI^+^ H22 cells was analyzed.

### 2.9. Immunohistochemistry Assay

Immunohistochemistry studies of T cells and CD8^+^ T cells in tumor tissues were performed as described previously [25]. Briefly, after the tumor tissues from model and LBP treatment group were fixed with 10% neutral formalin for 24 h, paraffin-embedded sections were prepared and stained with the purified antibodies of CD3 and CD8. The immunodetection was performed using a murine/rabbit IgG immunohistochemistry kit (Boster Biological Technology Co. Ltd., China) and SignalStain® DAB substrate kits (CST).

### 2.10. In Vitro Assay of the Effects of LBP on Cytokine Production in H22 Cells and RAW264.7 Macrophages

The H22 cells (1 × 10^4^ cells/well in 96-well plates) were treated with 200, 400, and 800 *μ*g/mL of LBP for 24 h. The RAW264.7 macrophages (1 × 10^5^ cells/well in 96-well plates) were treated with 25, 50, and 100 *μ*g/mL of LBP for 24 h. The control cells were treated with culture medium. The culture supernatants were collected to determine the level of TGF-*β*1 and IL-10 with ELISA kits.

### 2.11. Statistical Analysis

The data were expressed as mean ± SD. Student *t*-test was used to assess the statistical significance of differences between experimental groups. *P* value < 0.05 was considered statistically significant.

## 3. Results

### 3.1. LBP Treatment Inhibits Solid Tumor Growth, but Has Little Effect on Body Weight or Spleen Index in H22 Tumor-Bearing Mice

**Figure 1:**
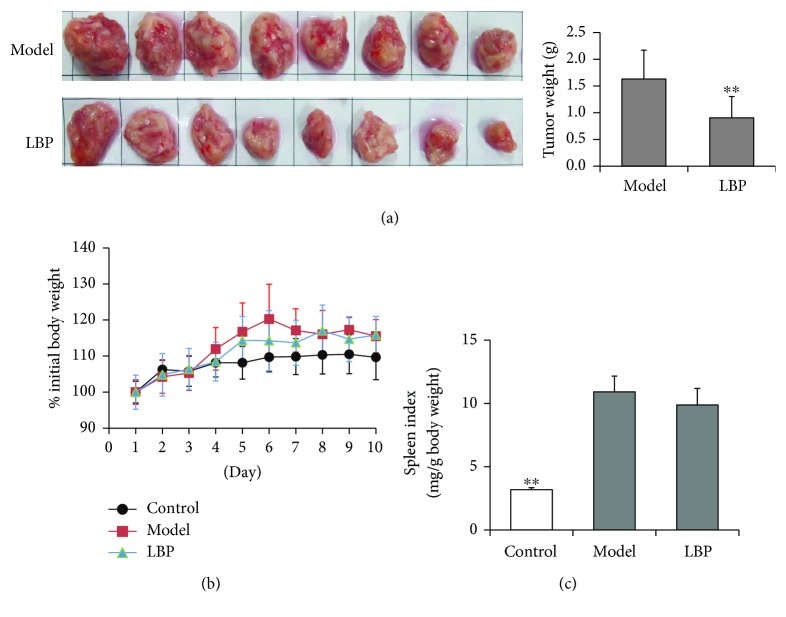
LBP treatment inhibited H22 solid tumor growth in mice. Mice were transplanted with H22 cells in the right armpit subcutaneously to prepare tumor-bearing mice. In LBP group, mice were treated with LBP (250 mg/kg) intragastrically for 10 days once daily. (a) Tumor weights from BALB/c mice. (b) Change of body weight. (c) Spleen index. Data are represented as mean ± SD, *n* = 8 in each group. ^∗∗^*P* < 0.01 versus model group.

Studies reported that LBP could inhibit tumor growth in mice [1–3]; also, our previous study demonstrated that the antitumor activity of LBP was closely related to its molecular weight and LBP with medium molecular weight (40–350 kDa) had the highest antitumor activity in H22 tumor-bearing mice [23]. In the present study, we further investigated the effect of such LBP on the systemic and local immune responses in H22 tumor-bearing mice. The tumor-bearing mice were injected subcutaneously with H22 cells into the right armpit. The tumor-free mice served as a control. The untreated tumor-bearing mice served as a model. Consistent with previous study, LBP inhibited H22 tumor growth dramatically in this study (Figure 1(a)). At the end of the experiment, LBP showed little effect on body weight or spleen index in mice when compared with those in the model group (Figures 1(b) and 1(c)). However, we found that the increase of body weight due to the tumor growth from day 4 to day 7 was lower in the LBP-treated mice than those in the model mice. From day 7 to day 10, the increase of body weight was decreased in the model mice compared with themselves on day 6 mainly due to the loss of muscle, while the increase of body weight always increased slowly in the LBP-treated mice since day 5. These results indicated that LBP not only inhibited H22 tumor growth but also reduced muscle loss in mice.

### 3.2. LBP Treatment Maintains High Levels of T Cells in PB, TDLN, and Tumor Tissue

**Figure 2:**
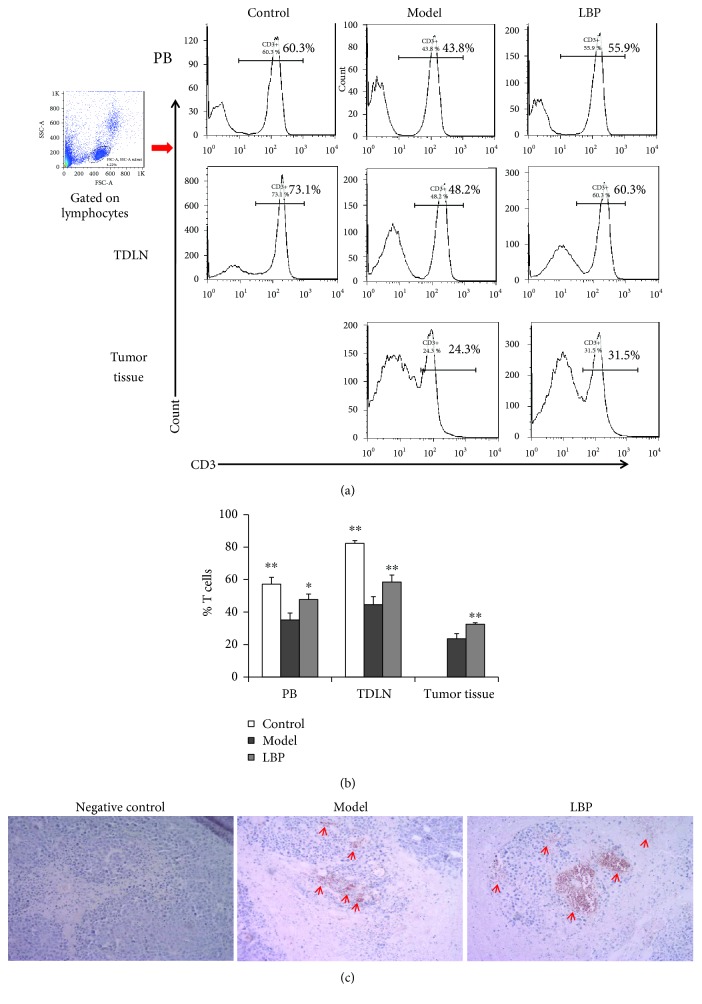
LBP treatment prevented the decrease of T cells in tumor-bearing mice. The percentage of T cells in PB, TDLN, and tumor tissue from tumor-free mice or H22 tumor-bearing mice was analyzed by FCM. The infiltration of T cells in tumor tissue was observed by immunohistochemistry. (a) Representative histograms of T cell percentage gated on lymphocytes. (b) Statistical analysis of the percentage of T cells. (c) Immunohistochemical analysis of T-cell infiltration (red arrows) in the tumor tissue (magnification 200x). Data are represented as mean ± SD, *n* = 8 in each group. ^∗^*P* < 0.05 and ^∗∗^*P* < 0.01 versus model group. PB: peripheral blood; TDLN: tumor draining lymph node; FCM: flow cytometry.

The presence of high levels of T cells in cancer patients is a favourable prognostic factor [13–15, 26], and adoptive T cell transfer for cancer has been proved to be a promise approach [27, 28]. Thus, T cell plays an important role in cancer treatment. In the present study, we investigated whether LBP affected T-cell level in H22 tumor-bearing mice. Lymphocytes from PB, TDLN, and tumor tissues were prepared, and the T-cell percentages in lymphocytes were determined with FCM. As shown in Figures 2(a) and 2(b), the tumor-bearing mice in the model group had lower T-cell percentages in PB and TDLN compared with those in the control group (*P* < 0.01). LBP-treated mice had higher percentages of T cells not only in PB and TDLN but also in tumor tissues, than those in the model group (*P* < 0.05 in PB, *P* < 0.01 in TDLN and tumor tissues). To confirm these results, we further investigated T-cell infiltration in tumor tissues with immunohistochemistry. Results from immunohistochemistry showed that more T-cell infiltration was observed in tumor tissue from LBP-treated mice than those from the model group (Figure 2(c)). These results demonstrated that LBP treatment prevented the decrease of T cells in both systemic and local tissues of the tumor-bearing mice.

### 3.3. LBP Treatment Prevents the Increase of CD4^+^CD25^high^ Tregs While Promotes Infiltration of CD8^+^ T Cells in Tumor Tissue

**Figure 3:**
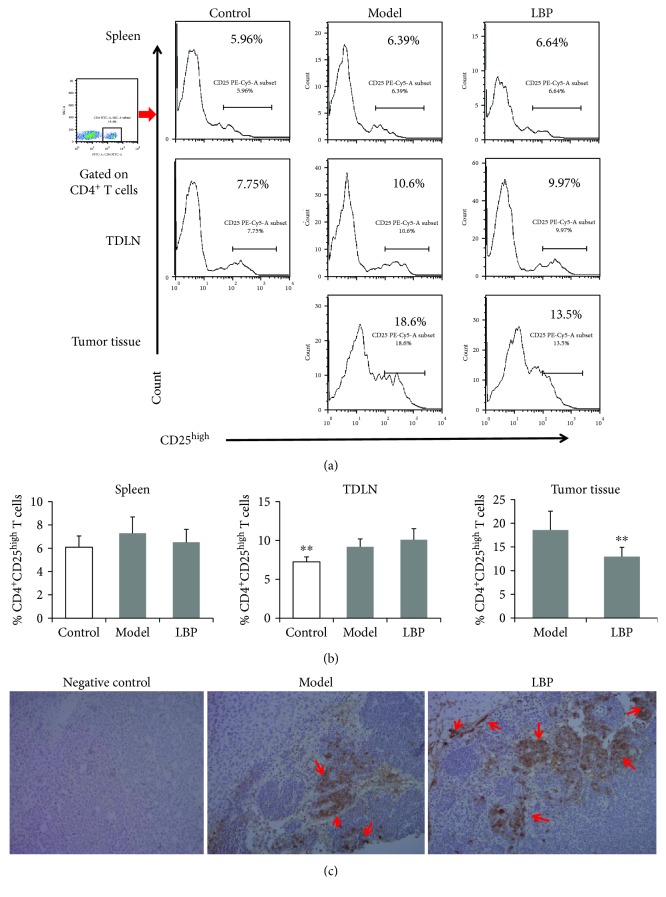
LBP treatment prevented the increase of CD4^+^ CD25 ^high^ Tregs while promoted the infiltration of CD8^+^ T cells in tumor tissue. (a) Representative dot plots of CD4^+^ CD25 ^high^ Tregs percentages gated on CD4^+^ T cells in TDLN, spleen, and tumor tissue. (b) Statistical analysis of the percentage of CD4^+^ CD25 ^high^ Tregs in TDLN, spleen, and tumor tissue. (c) Immunohistochemical analysis of CD8 (red arrows) in the tumor tissue (magnification 200x). Data are represented as mean ± SD, *n* = 8 in each group. ^∗∗^*P* < 0.01 versus model group. TDLN: tumor draining lymph node.

As mentioned above, LBP maintained high levels of T cells in the tumor-bearing mice. However, there are two major T-cell subsets, including CD8^+^ cytotoxic T lymphocytes (CTL) and CD4^+^ regulatory T cells (Tregs), which play an opposite role in tumor immunity [19, 29], respectively. Tregs can promote tumor growth, while CTL can kill the cancer cells. Thus, in the present study, we further investigated whether LBP affected distribution of Tregs and CD8^+^ T cells in the tumor-bearing mice. Lymphocytes from the spleen, TDLN, and tumor tissues were prepared as described above. The percentages of Tregs in T cells were determined with FCM, while the infiltration of CD8^+^ T cells in tumor tissue was assayed with immunohistochemistry. Since most of the CD4^+^ Tregs can be identified by the high expression of CD25 molecules on their surface [30], CD4^+^CD25^high^ T cells were distinguished as Tregs in this study. As shown in Figure 3, the tumor-bearing mice had higher percentages of Tregs in TDLN (*P* < 0.01) and spleen (slightly upregulated, but not significantly) than those in the control group. Compared with the model group, LBP treatment showed little effect on the level of Tregs in TDLN or spleen; however, it could significantly reduce Tregs in tumor tissue (*P* < 0.05 when compared with the model group, Figures 3(a) and 3(b)). The results indicated that LBP might increase the infiltration of CD8^+^ T cells in tumor tissue, because Tregs have the ability to inhibit the proliferation of CD8^+^ T cells. We therefore further investigated the effect of LBP on the infiltration of CD8^+^ T cells in the tumor tissue. As expected, the results in immunohistochemistry showed that LBP-treated mice had more infiltration of CD8^+^ T cells in tumor tissue than those in the model group (Figure 3(c)). Our results demonstrated that LBP treatment prevented the increase of CD4^+^ CD25^high^ Tregs and promoted infiltration of CD8^+^ T cells in tumor tissue.

### 3.4. LBP Treatment Inhibits the Production of TGF-*β*1 and IL-10 in Tumor-Bearing Mice

**Figure 4:**
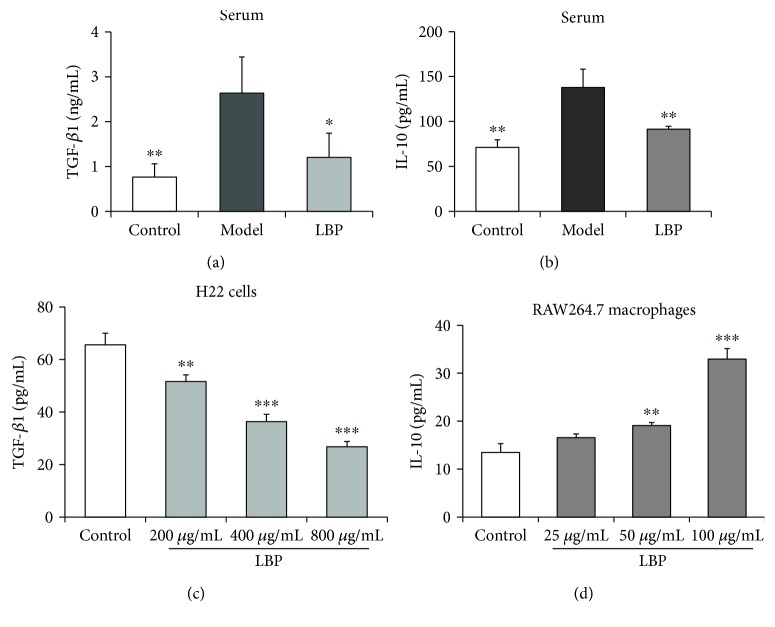
LBP treatment inhibited the production of TGF-*β*1 and IL-10 in H22 tumor-bearing mice. Effects of LBP on the production of TGF-*β*1 and IL-10 in the serum of the mice and culture supernatants of H22 cells and RAW264.7 macrophages were investigated by ELISA kits. (a) The level of TGF-*β*1 in serum. (b) The level of IL-10 in serum. (c) Effect of LBP on TGF-*β*1 production in H22 cells. (d) Effect of LBP on IL-10 production in RAW264.7 macrophages. Data are represented as mean ± SD, *n* = 8 in each group of the mice, *n* = 3 in each group of the cells. ^∗^*P* < 0.05 and ^∗∗^*P* < 0.01 versus model group in mice; ^∗∗^*P* < 0.01 and ^∗∗∗^*P* < 0.001 versus control group in the cells. ELISA: enzyme-linked immunosorbent assay.

TGF-*β*1 and IL-10 play important roles in the development and suppressive function of Tregs [31, 32]. To illuminate how LBP prevents the increase of Tregs in the tumor-bearing mice, we therefore investigated whether LBP could affect the production of TGF-*β*1 and IL-10 in mice. We first investigated the levels of TGF-*β*1 and IL-10 in serum with ELISA kits. As shown in Figure 4, the tumor-bearing mice in the model group had higher levels of TGF-*β*1 (Figure 4(a)) and IL-10 (Figure 4(b)) in serum than those in the control group (*P* < 0.01), while LBP-treated mice had lower levels of such cytokines in serum when compared with model mice (*P* < 0.05 and *P* < 0.01, resp.). These results demonstrated that LBP could inhibit the production of TGF-*β*1 and IL-10 in H22 tumor-bearing mice, which might contribute to the prevention of Tregs function in LBP-treated mice.

Macrophages and some cancer cells can produce TGF-*β*1 and IL-10 [31, 33, 34]. To illuminate how LBP inhibits the production of TGF-*β*1 and IL-10 in H22 tumor-bearing mice, we then investigated the effects of LBP on TGF-*β*1 and IL-10 production in H22 cells and RAW264.7 macrophages *in vitro*. The cells were treated with LBP for 24 h, and the culture supernatants were collected and assayed. The results showed that H22 cells could produce TGF-*β*1, but not IL-10; LBP could inhibit the production of TGF-*β*1 in H22 cells in a dose-dependent manner (Figure 4(c)). However, LBP could not inhibit the production of IL-10 in RAW264.7 macrophages; on the contrary, LBP could promote the IL-10 section (Figure 4(d)). These results demonstrated that LBP inhibited the production of TGF-*β*1 in the H22 tumor-bearing mice partly by inhibiting TGF-*β*1 secretion in H22 cells. Meanwhile, the inhibition of IL-10 production in LBP-treated mice was probably due to the inhibition of Tregs, because Tregs can also produce IL-10.

### 3.5. LBP Treatment Decreases the Exhaustion Phenotype of T Cells in Tumor-Bearing Mice

**Figure 5:**
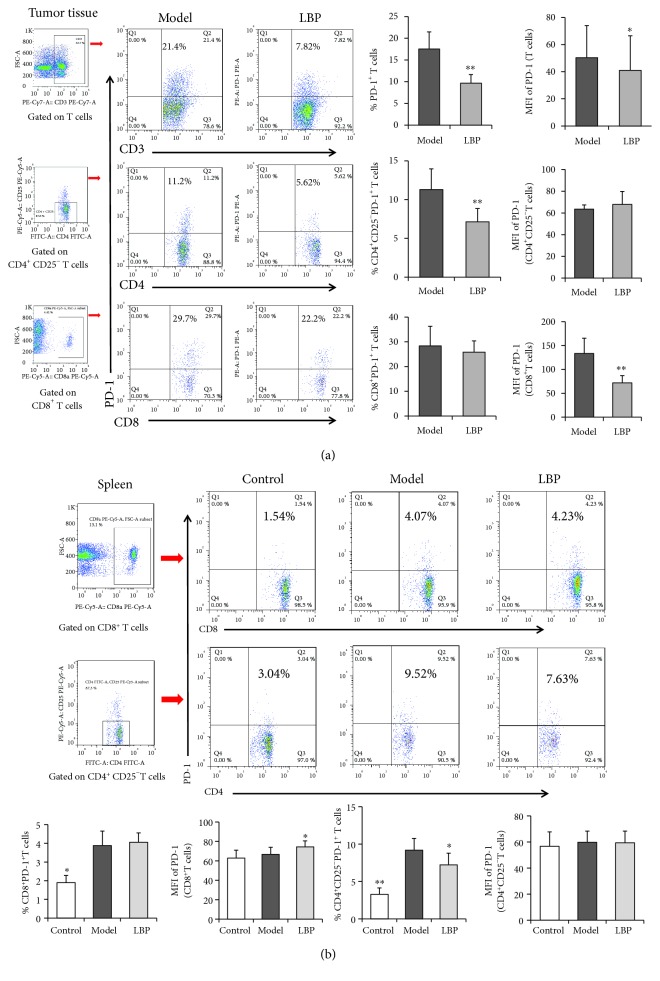
LBP treatment inhibited the expression of PD-1 on T-cell subsets. The PD-1 expression on T cells in tumor tissue and spleen was analyzed by FCM. (a) PD-1 expression on T cells, CD4^+^ CD25^−^ T cells and CD8^+^ T cells in tumor tissue. (b) PD-1 expression on CD4^+^ CD25^−^ T cells and CD8^+^ T cells in the spleen. PD-1 expression on T cell subsets was statistical analysis as the percentage and MFI, simultaneously. Data are represented as mean ± SD, *n* = 8 in each group. ^∗^*P* < 0.05 and ^∗∗^*P* < 0.01 versus model group. PD-1: programmed cell death-1; FCM: flow cytometry; MFI: mean fluorescence intensity.

Though LBP promoted the infiltration of T cells in the tumor tissues, previous study reported that the tumor-infiltrating T cells are exhausted [11]. PD-1 expression is markedly upregulated on tumor-infiltrating T cells, especially on CD8^+^ T cells, which has been demonstrated as T cell exhaustion phenotype [11, 35]. To investigate whether LBP affected the T cell exhaustion in the tumor-bearing mice, we analyzed the PD-1 expression on effector T cells—CD4^+^ CD25^−^ T cells and CD8^+^ T cells in tumor tissues and spleens using the data of percentage and mean fluorescence intensity (MFI) which indicates the number of PD-1 on cell surface. The results showed that LBP-treated mice had lower percentage and MFI of PD-1 expression on T cells in tumor tissues than those in the model group (*P* < 0.05 and *P* < 0.01, respectively, Figure 5(a)). Further analysis showed that LBP-treated mice had lower MFI of PD-1 on CD8^+^ T cells and percentage of CD4^+^ CD25^−^ PD-1^+^ T cells in tumor tissues than those in the model group (*P* < 0.01). The tumor-bearing mice in the model group had higher percentages of CD8^+^ PD-1^+^ T cells and CD4^+^ CD25^−^ PD-1^+^ T cells in the spleens than those in the control group. Compared with the mice in the model group, LBP-treated mice had higher MFI of PD-1 on CD8^+^ T cells and lower percentages of CD4^+^ CD25^−^ PD-1^+^ T cells in the spleens (*P* < 0.05, Figure 5(b)). These results demonstrated that LBP treatment could decrease the exhaustion phenotype of T cells in H22 tumor-bearing mice.

### 3.6. LBP Treatment Maintains the Cytotoxicity of Lymphocytes in TDLN and Spleen

**Figure 6:**
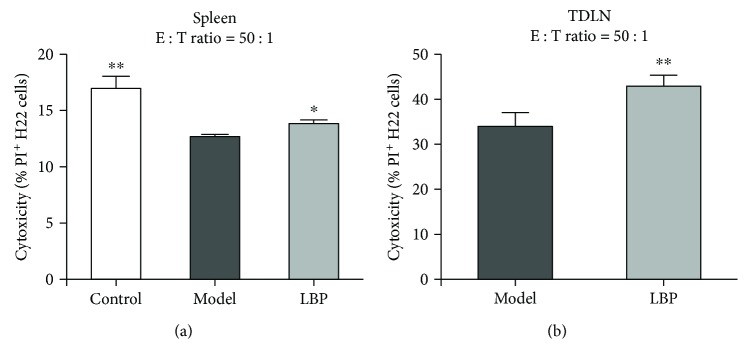
LBP treatment maintained cytotoxicity of lymphocytes from TDLN and spleen. H22 cells as target cells were labeled with CFSE before incubation with lymphocytes. The effector cells (lymphocytes) and target cells (H22 cells) were incubated for 24 h at a ratio of 50 : 1. After staining of PI, FCM was performed to calculate the percentage of PI^+^ H22 cells. (a) The percentage of H22 cells killed by lymphocytes from the spleen. (b) The percentage of H22 cells killed by lymphocytes from TDLN. Data are represented as mean ± SD, *n* = 8 in each group. ^∗^*P* < 0.05 and ^∗∗^*P* < 0.01 versus model group. CFSE: carboxyfluorescein diacetate succinimidyl ester; PI: propidium iodide; FCM: flow cytometry; TDLN: tumor draining lymph node.

The cytotoxicity of lymphocytes against cancer cells is a powerful antitumor immune response. CD8^+^ CTL and NK cells are two types of lymphocytes that can kill the cancer cells in a cytotoxic manner. As shown above, LBP treatment decreased the exhaustion phenotype of T cells and the infiltration of Tregs in tumor tissues as well as the production of suppressive cytokines. These could contribute to maintain the antitumor immune responses in H22 tumor-bearing mice. Thus, we then further investigated the effects of LBP on the cytotoxicity of lymphocytes against H22 cells. Lymphocytes in TDLN and spleens of the mice were separated and cultured with H22 cells for 24 h. H22 cells that were killed by lymphocytes were determined using FCM. As shown in Figure 6, the results showed that lymphocytes in the spleens from the model group had lower cytotoxicity to H22 cells than those in the control group (*P* < 0.01). Compared with the model group, lymphocytes in the spleens and TDLN from the LBP treatment group had higher cytotoxicity to H22 cells (*P* < 0.05 in the spleen, *P* < 0.01 in TDLN). These results demonstrated that LBP treatment maintained cytotoxicity of lymphocytes against H22 cells in the H22 tumor-bearing mice.

## 4. Discussion

In the present study, we investigated the effects of LBP on systemic and local immune responses in H22 tumor-bearing mice. We found that LBP could maintain high levels of T cells in systemic and local tissues, prevent the increase of Tregs while promoting infiltration of CD8^+^ T cells in tumor tissue, inhibit the production of TGF-*β*1 and IL-10 in serum, decrease the exhaustion phenotype of T cells, and maintain cytotoxicity of lymphocytes. These results demonstrated that LBP treatment simultaneously induced systemic and local T cell-dependent antitumor immune responses in H22 tumor-bearing mice.

As known, T cells play an important role in cancer prevention. The presence of high level of T cells is a favourable prognostic factor in human tumors [13–15]. Studies have shown that adoptive T-cell therapy is a potential powerful approach for developing safe and effective cancer therapeutics [27, 28]. Though the antitumor and immune regulation activity of LBP had been demonstrated, the effect of LBP on phenotypic and functional change of T cells is still largely unknown. In this study, we found that LBP treatment prevented the decrease of T cells in PB, TDLN, and tumor tissue of H22 tumor-bearing mice. One possible reason for these results might be that LBP promoted the activation and proliferation of T cells in H22 tumor-bearing mice. Evidence supported our conclusion wherein LBP could promote T-cell proliferation *in vitro* and *in vivo* [20, 21, 36].

However, accumulated evidence has shown that T cells play a duplicitous role in cancer due to the existence of different T-cell subsets. T cells can be subdivided into two major cell types on the basis of functional difference in tumor immunity—Tregs and effector T cells that they can promote or inhibit tumor growth, respectively [11, 12]. Tregs are one type of T cells that can actively suppress the antitumor immune responses and promote tumor growth [19]. Tregs suppress the antitumor immune response by expression of coinhibitory molecules on surface and secretion of suppression cytokines, such as CTLA-4, IL-10, and TGF-*β*1 [37–39]. Most of the Tregs can be distinguished by the high expression of CD25 and CD4 on their surface which has become a target for Tregs depletion and sorting [19, 40–42], although the expression of internal transcription factor Foxp3 is another characteristic for Tregs identification. Studies reported that increased Tregs infiltration in some tumors is associated with poor survival [16–18]. Depletion of Tregs or blockade of the coinhibitory molecules on Tregs surface can reverse the imbalance between pro- and antitumor immunities [19, 43]. Therefore, Tregs have become a target in cancer immunotherapy. In this study, we found that LBP treatment inhibited the increase of CD4^+^ CD25^high^ Tregs in tumor tissue, as well as the secretion of IL-10 and TGF-*β*1 in serum. IL-10 and TGF-*β*1 are not only immunosuppressive cytokines but also play a critical role in the development and suppressor function of Tregs [31, 32]. Besides, IL-10 and TGF-*β*1 are also produced by other cell types, such as macrophages and some cancer cells [31, 33, 34]. Our results showed that LBP could inhibit TGF-*β*1 secretion in H22 cells, but could not inhibit IL-10 secretion in RAW264.7 macrophages. These results indicated that LBP inhibited the production of TGF-*β*1 partly by inhibiting TGF-*β*1 secretion in H22 cells, which contributed to decrease Tregs in mice. Meanwhile, the decrease of Tregs could contribute to the production of IL-10 in LBP-treated mice.

Effector T cells are composed of CD8^+^ CTL and CD4^+^ Th1 cells. One of major mechanisms that the CD8^+^ CTL inhibits tumor growth is by killing the tumor cells in a cytotoxic manner, while CD4^+^ Th1 cells may work by secreting cytokines. In this study, we used the immunohistochemistry to detect the infiltration of CD8^+^ T cells in tumor tissue. We found that LBP treatment could improve the infiltration of CD8^+^ T cells in tumor tissue which often indicates the good prognosis. However, accumulated studies have shown that most T cells in tumor microenvironment, especially CD8^+^ T cells, are exhausted with high PD-1 expression [11, 30]. We found that LBP treatment inhibited the PD-1 expression on T-cell subsets, including CD4^+^CD25^−^ and CD8^+^ T cells in tumor tissue. This indicated that LBP treatment decreased the exhaustion phenotype of T cells. However, we also found that LBP treatment inhibited the proportion of CD4^+^ CD25^−^ PD^+^ T cells while increased the PD-1 expression level on CD8^+^ T cells in the spleen. These indicate that multiple mechanisms may be responsible for LBP in regulating PD-1 expression on CD8^+^ T cells between system and tumor microenvironment. In fact, previous study showed that CD8^+^ PD-1^+^ T cells were exhausted in tumor and functional in draining lymph nodes of colorectal cancer patients [35].

The results as mentioned above that LBP treatment decreased the exhaustion phenotype of T cells and the infiltration of Tregs in tumor tissues as well as the production of suppressive cytokines could contribute to enhance the antitumor immune response in tumor-bearing mice. In order to demonstrate this conclusion, we further investigated the cytotoxicity of lymphocytes in TDLN and spleen against H22 cells. As expected, LBP treatment could maintain the cytotoxicity of lymphocytes in TDLN and spleen. This was consistent with previous study that LBP could enhance CTL activity in S180 tumor-bearing mice [2]. In fact, CD8^+^T cells and NK cells are the lymphocytes in TDLN and spleen that could kill the cancer cells in a cytotoxicity manner, while Tregs can suppress their antitumor activity [44–46]. Therefore, the possible mechanisms that LBP maintains the cytotoxicity of lymphocytes in our study might be by inhibiting of Tregs function and preventing T cells from exhaustion.

## 5. Conclusions

Taken together, our results demonstrated that LBP successfully induced systemic and local T cell-dependent antitumor immune responses in H22 tumor-bearing mice mainly by alleviating immunosuppression and maintaining antitumor immune responses. Since immunosuppressive tumor microenvironment is a big obstacle in cancer immunotherapy, our results indicate that LBP may be an effective and ideal reagent for cancer treatment in combining with immunotherapy, especially with adoptive cellular immunotherapy.
